# Dosimetric Feasibility of Tomotherapy-Based Selective Axillary Sparing Regional Nodal Irradiation for Lymphedema Risk Reduction in Breast Cancer

**DOI:** 10.3390/medicina61122177

**Published:** 2025-12-07

**Authors:** Kwang Hwan Cho, Cheol Wan Lim, Sung-Mo Hur, Zisun Kim, Jae-Hong Jung, Daegun Kim, Seung-Gu Yeo

**Affiliations:** 1 Department of Radiation Oncology, Soonchunhyang University College of Medicine, Soonchunhyang University Hospital, Bucheon 14584, Republic of Korea; medphy@schmc.ac.kr (K.H.C.); rtjung@schmc.ac.kr (J.-H.J.); hidegun@schmc.ac.kr (D.K.); 2 Department of Surgery, Soonchunhyang University College of Medicine, Soonchunhyang University Hospital, Bucheon 14584, Republic of Korea; cwlim@schmc.ac.kr (C.W.L.); smkine@schmc.ac.kr (S.-M.H.); zskim@schmc.ac.kr (Z.K.)

**Keywords:** breast cancer, regional nodal irradiation, lymphedema, axillary lateral vessel thoracic junction, Tomotherapy

## Abstract

*Background and Objectives*: The axillary lateral vessel thoracic junction (ALTJ) is a key lymphatic drainage pathway for the arm and a potential structure to spare during regional nodal irradiation (RNI) to reduce lymphedema risk in breast cancer patients. This study aims to demonstrate the feasibility of ALTJ-sparing radiation therapy (RT) planning using Tomotherapy. *Materials and Methods*: Ten breast cancer patients who had undergone axillary lymph node dissection and whose dissected axillary levels were excluded from the RNI target volume were included. A TomoDirect intensity-modulated RT plan was generated at a dose of 50 Gy in 25 fractions. The dissected axilla was not designated as an organ at risk (OAR) in the original treatment plan. For this study, the axillary lymph node level I (AXL1) and the ALTJ were delineated retrospectively, with the ALTJ considered an OAR in the newly generated study plan. A total of 20 RT plans (10 per group) were statistically compared using various dose-volume parameters. *Results*: Compared to the original plans, the study plans with ALTJ as an OAR significantly reduced the incidental dose to both the ALTJ (mean: 41.7 ± 3.4 Gy vs. 27.2 ± 1.3 Gy; *p* = 0.005) and the AXL1 (mean: 43.9 ± 2.0 Gy vs. 37.7 ± 1.9 Gy; *p* = 0.005). All other dosimetric parameters (V25Gy, V35Gy, V40Gy, Dmin, Dmax) for the ALTJ were also significantly lower in the study plans. This ALTJ sparing was achieved while maintaining all required dose-volume constraints for target volumes and standard OARs such as the lung and heart. *Conclusions*: This study demonstrates that simply excluding the dissected axilla from the target volume without designating it as an OAR still results in a substantial incidental dose to this region. Our findings also show the feasibility of using Tomotherapy to selectively spare the axilla, particularly the ALTJ subregion of AXL1, which is critical for lymphedema risk in breast cancer patients.

## 1. Introduction

Postoperative adjuvant radiation therapy (RT) including regional nodal irradiation (RNI) plays a critical role in reducing locoregional recurrence and improving disease-free survival in breast cancer, particularly in patients with node-positive disease or other high-risk pathological features [1]. However, after combined multi-modality therapies, ipsilateral upper extremity lymphedema remains a common and burdensome late toxicity for breast cancer patients, with reported incidence rates of 15–30% depending on the extent of surgery and use of adjuvant therapies [2]. Axillary lymph node dissection (ALND) and RNI are together estimated to account for up to 75% of breast cancer-related lymphedema, with ALND being the predominant contributor [3]. This underscores the importance of minimizing radiation exposure to the axilla in patients with prior ALND, who already have an elevated baseline risk.

Using axillary reverse mapping (ARM), it has been reported that in 92% of the 172 breast cancer patients, the lymph nodes (LNs) draining the arm was localized to the superior-lateral axilla, which corresponds to the axillary lateral vessel thoracic junction (ALTJ) region [4]. Additionally, it has been shown that the ARM nodes and breast-draining sentinel nodes are distinct LNs in the vast majority of cases [5]. These studies highlight the ALTJ—a subregion within axillary LNs level I—as a key component of arm lymphatic drainage and support its consideration as a potential critical structure for selective sparing during RNI.

Moving beyond conventional field-based RT techniques, target volume contouring based on anatomical definitions is becoming a standard practice in breast cancer [6]. This approach allows for individualized RT planning and optimization of radiation dose distribution while minimizing doses to organs at risk (OARs). Tomotherapy, a unique form of intensity-modulated RT (IMRT), enables continuous delivery without the need for field matching during whole breast or chest wall irradiation with RNI, thereby eliminating the risk of dose hotspots at junction lines—which is particularly important for regions like the ALTJ that lie near conventional field borders [7].

In this study, we evaluated the dosimetric feasibility of Tomotherapy-based selective axillary sparing during RNI for post-ALND breast cancer patients.

## 2. Materials and Methods

A total of 10 breast cancer patients who underwent adjuvant RT with RNI between 2024 and 2025 were included in this study. Six of these patients had breast-conserving surgery, while the remaining four had modified radical mastectomy. The laterality of breast cancer was equally distributed. They had undergone ALND (with ≥10 LNs resected) and were deemed to have low residual nodal risk, so the dissected axillary levels (level I to the lateral portion of level II) were excluded from their RNI target [1,8].

For simulation, patients were immobilized on a breast-tilting board with both arms abducted above the head. Axial computed tomography (CT) slices, obtained at 3 mm intervals using SOMATOM Confidence (Siemens Healthcare, Erlangen, Germany), were then transferred to MIM Maestro (version 6.6, MIM Software, Cleveland, OH, USA) for contouring the regions of interest. The clinical target volume (CTV) of the breast or chest wall, and the ipsilateral axillary nodal levels from II to IV, were contoured separately, with or without internal mammary nodes, according to the European Society for Radiotherapy and Oncology contouring guidelines [9]. The planning target volume (PTV), a single contiguous volume for the breast or chest wall and regional nodes, was created by expanding the CTV by 5 mm in all directions and cropping 3 mm from the skin surface. The standard OARs included the ipsilateral and contralateral lungs, the heart, the contralateral breast, the spinal cord, and the ipsilateral humerus head. The dissected axilla was not designated as an OAR in the plan used for patient treatment. The axilla level I (AXL1) and the ALTJ, the latter based on guidelines published by Gross et al. [10], was delineated retrospectively for this study by one experienced radiation oncologist (S-G Y). The ALTJ was defined cranially by one axial slice below the lowest contour of the humeral head and caudally by the inferior-most contour of the axillary vessels, while its anterior border is the plane defined by the latissimus dorsi and pectoralis musculature, its posterior border is the anterior surface of the subscapularis muscle, its lateral border includes the axillary vessels, and its medial border is the lateral border of the pectoralis minor muscle.

A TomoDirect IMRT plan was generated at a dose of 50 Gy in 25 fractions using Accuracy Precision (version 3.3.1.3, Accuray Inc., Sunnyvale, CA, USA). The plan was optimized to meet the dose-volume constraints of the standard OARs, with the PTV normalized to receive at least 95% of the prescription dose in 95% of its volume and to a maximum dose (Dmax) of no more than 107%. For the ipsilateral lung, the dose-volume constraints were V20Gy ≤ 40%, V10Gy ≤ 65%, and V5Gy ≤ 75%. The contralateral lung was constrained to V5Gy ≤ 15%. For patients with left-sided breast cancer, the mean heart dose was limited to ≤5 Gy, with V30Gy ≤ 10% [11]. Referring to data from previously published studies [10,12,13], the dose-volume constraints for the ALTJ were set as V22Gy ≤ 70%, V27Gy ≤ 35%, and V32Gy ≤ 10%. Using four binary multi-leaf collimator leaves, the field size was expanded with a 25.0 mm margin on the outer edges to cover the flash region. The treatment planning parameters included a field width of 5.0 cm in dynamic jaw mode, a pitch of 0.5, and a modulation factor of 2.4. The 6-MV photon beam was used for each field.

For each patient, two different treatment plans were compared, one of which was newly created for this study. The first conventional plan, used for patient treatment, utilized a total of four beams, consisting of two tangential beams and two additional anterior beams, with only standard OARs designated. The second study plan designated the ALTJ as an additional OAR and, in addition to the four beams from the first plan, an extra beam was added at an angle optimized to maximally spare the ALTJ.

For each plan, a Dose-Volume Histogram (DVH) was constructed. The incidental dose to the AXL1 and ALTJ in the conventional plan was first analyzed. Subsequently, a comparative analysis of dose-volume parameters for the ALTJ, AXL1, and standard OARs was performed between the conventional and study plans. Dosimetric analysis included several parameters, such as the percentage volume receiving a specific dose (Gy), the mean dose (Dmean), minimum dose (Dmin), and Dmax. The two groups were compared using the Wilcoxon signed-rank test, with a *p*-value of <0.05 considered statistically significant. Statistical analysis was carried out using SPSS software (version 30.0, SPSS Inc., Chicago, IL, USA).

## 3. Results

The patients consisted of 6 who received whole breast plus RNI and 4 who received chest wall plus RNI, two of whom also had IMN included as part of their RNI. A total of 20 Tomotherapy IMRT plans were analyzed using DVHs. The mean volumes for the contoured ALTJ and AXL1 were 8.6 ± 2.4 mL and 49.5 ± 11.9 mL, respectively. The AXL1 volume had some overlap with the CTV to PTV margin.

In the conventional treatment plans, the incidental radiation dose to the ALTJ averaged 41.7 ± 3.4 Gy, with a V35Gy of 82.7 ± 13.5%. Similarly, the incidental dose to the AXL1 in the conventional plans averaged 43.9 ± 2.0 Gy, with a V35Gy of 87.5 ± 7.6%. In the study plans, where the ALTJ was included as an OAR, the mean dose to the ALTJ was reduced to 27.2 ± 1.3 Gy, with a V35Gy of 9.6 ± 4.7%. For the AXL1, which encompasses the ALTJ, the mean dose was 37.7 ± 1.9 Gy, and the V35Gy was 64.5 ± 6.2%.

The results of statistical analysis showed that all analyzed dosimetric parameters for the ALTJ were significantly lower in the study plans compared to the conventional plans (Table 1). For the AXL1, all dosimetric parameters were significantly lower in the study plans, with the exception of Dmin and Dmax, which showed no significant differences. Although the standard OARs showed no significant differences between the two plans, certain parameters were lower in the study plans (Table 2). This may be due to the introduction of additional beam angles in the study plan, but the small absolute differences and limited sample size necessitate further investigation.

**Figure 1:**
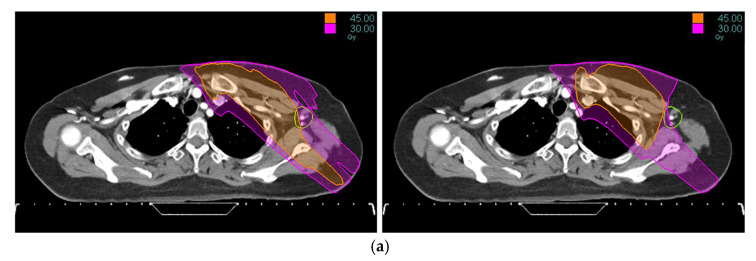

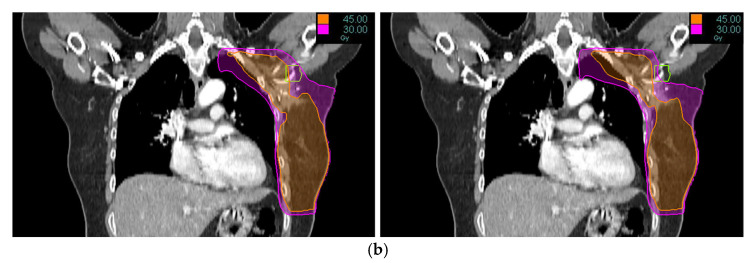
Tomotherapy plan image of a typical patient: (**a**) axial view, (**b**) coronal view. The contoured ALTJ (green) is shown, and the dose distributions with isodose lines (30 and 45 Gy) compared between conventional (**left**) vs. study plan (**right**).

**Figure 2:**
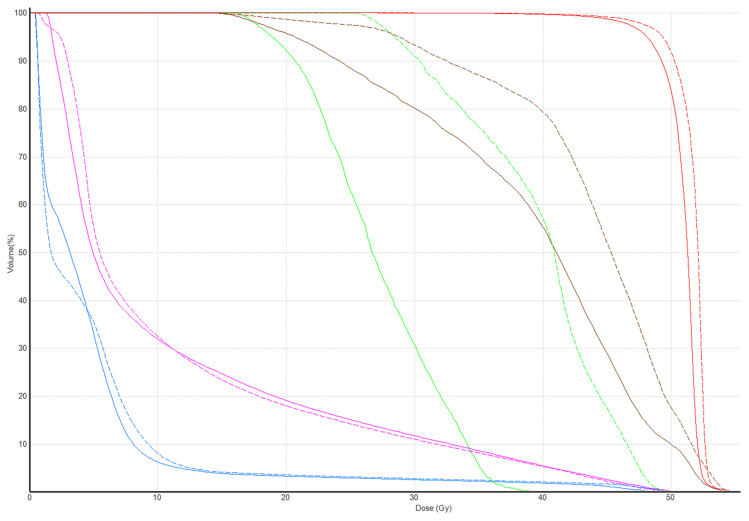
DVH of the patient in Figure 1. Dose-volume lines of PTV (red), ALTJ (green), AXL1 (brown), ipsilateral lung (magenta), and heart (blue) compared between conventional (dashed line) vs. study plan (solid line).

Tomotherapy plan images along with the DVH of a typical patient is illustrated in Figure 1 and Figure 2. The newly generated plans, successfully optimized to meet all standard dose-volume constraints for both target volumes and standard OARs, achieved significant sparing of the ALTJ without compromising the dose received by other critical OARs.

## 4. Discussion

Recent clinical guidelines are increasingly supporting a selective approach to RNI after ALND [14,15], noting that previously dissected axillary levels can be excluded from the CTV. Nevertheless, these guidelines do not explicitly recommend treating these areas as OARs, which leaves IMRT planning strategies open to further optimization. In a previous investigation of TomoDirect hypofractionated whole breast irradiation [16], we observed a low incidental dose of axillary level I (mean 15.5 Gy, corresponding to an equivalent dose in 2 Gy fractions of 17.5 Gy_3_) in patients for whom RNI was not indicated. In the present study, however, the incidental dose to axillary level I, a volume that includes the ALTJ subregion, was substantially higher in patients who required RNI {mean: 87.8% (43.9 Gy) of prescribed dose in the present study vs. 36.5% (15.5 Gy) of prescribed dose in a prior study [16]}. Ladbury et al. also reported that a substantial radiation dose is incidentally administered to the dissected axilla, despite its exclusion from the RNI target volumes [17]. This is likely attributable to the inclusion of levels II-IV in the target volume, which leads to a broader anatomical proximity of level I to the target volume. In addition, this happens because lung and heart sparing take planning priority, and the dissected axilla is not specifically recognized as a structure to avoid. Therefore, our results show that simply excluding the dissected axilla from the CTV without designating it as an OAR may still result in substantial radiation dose to this region. Moreover, the selective designation of the ALTJ as an OAR in Tomotherapy achieved a meaningful dose reduction to a highly susceptible subregion of AXL1 while preserving the dose sparing of standard OARs.

Similar to our own, the planning study by Waldstein et al. aimed to spare arm-draining LNs [18]. The main difference was that their RNI encompassed entire axillary levels including AXL1. However, unlike our approach, which designates a portion of an excluded region as an OAR, designating a subregion within the intended treatment volume as an OAR necessitates greater caution from an oncologic safety perspective. It should be considered that there is a higher likelihood for cross-drainage between breast and arm lymphatic pathways as the burden of nodal disease increases [10].

Gross et al. retrospectively contoured eight distinct axillary subregions on the planning CT scans of 265 breast cancer patients [10]. In this group of patients, mastectomy was performed in 55.1% of patients, and the median number of LNs removed was 11. The overall cumulative incidence of lymphedema was 15.6% at 3 years. This study demonstrated significant associations between dosimetric variables of the majority of axillary subregions and lymphedema, with the ALTJ exhibiting the strongest dosimetric association among them. The most significant dosimetric variable and cut point was an ALTJ Dmin of <36.8 Gy, which was associated with a 6.6-fold decrease in 3-year lymphedema rates (5.7% vs. 37.4%).

Chang et al. conducted an analysis of 1449 breast cancer patients [12]. In this group, axillary surgery involved removing six or fewer LNs in 59.5% of cases, and RNI was used in 32.5% of patients. The median follow-up time was 77.3 months, and the 5-year lymphedema rate was 6.8%. The ALTJ was retrospectively delineated, and both dosimetric and clinical parameters were analyzed. The lowest lymphedema rate (5-year, 1.2%) was observed in patients with ≤6 removed LNs and ≤66% ALTJ V35Gy. The highest lymphedema rate (5-year, 71.4%) was observed in patients with >15 removed LNs and an ALTJ Dmax of >53 Gy. In their second analysis, the number of LNs dissected and ALTJ V35Gy were found to be the most important factors influencing lymphedema [13]. These results underscore the potential value of ALTJ dose-volume constraints and support its consideration as an OAR in breast cancer RT planning.

Conflicting evidence has also been reported, suggesting no significant association between ALTJ dose and lymphedema. Healy et al. retrospectively contoured the ALTJ in 378 breast cancer patients; 71% underwent mastectomy, and a median of 18 axillary nodes were removed [19]. The 5-year cumulative incidence of lymphedema was 25.8%. On multivariate analysis, none of the ALTJ metrics were associated with lymphedema risk. Only increasing age, increasing body mass index, and the number of removed nodes were associated with a higher risk of developing lymphedema. This finding suggests that the ALTJ may not always represent the sole or primary arm drainage pathway.

This discrepancy may be partly explained by collateral lymphatic development that occurs after ALND. Using a retrospective review of a lymphoscintigraphy database, Fanning et al. compared the functional drainage of the upper extremity lymphatic system between patients with upper extremity cutaneous melanoma who had a prior ALND for cancer treatment but did not develop lymphedema and those without a prior ALND, to elucidate altered pathways of lymphatic drainage from the upper extremity following ALND [20]. The arm-draining sentinel LNs were categorized anatomically into axillary levels I–III, brachial, epitrochlear, and other locations. Compared with the control group, the ALND group showed a markedly reduced proportion of level I sentinel LNs (27% vs. 98%) but significantly higher proportions of sentinel LNs in levels II (27% vs. 3%) and III (32% vs. 1%), as well as increased brachial and epitrochlear drainage. In other lymphoscintigraphy studies [21,22], it was shown that breast cancer patients who underwent ALND exhibited a higher frequency of redirected arm-draining lymphatic flow to alternative extra-axillary routes compared to those who underwent sentinel LN biopsy. These findings suggest that after more aggressive axillary surgery, collateral lymphatic pathways develop, including drainage to preserved axillary levels II-III and to deep lymphatic systems, which may help mitigate the risk of lymphedema. In the three aforementioned ALTJ studies, the patients in the study [19] that showed no association between ALTJ dose and lymphedema had a relatively higher number of dissected axillary LNs compared with the other two studies [10,12].

In contrast to surgical procedures, where the ARM nodes can be directly visualized and selectively spared intraoperatively [23,24], postoperative RT planning relies on fixed anatomical coordinates from simulation CT and cannot account for dynamic postoperative lymphatic changes. Early mapping studies that used contralateral arm injections before surgery to approximate the ipsilateral ARM node location are of limited use for post-ALND cases [25]. Experimental and anatomical studies demonstrate that collateral lymphatic pathways can form within days and undergo significant remodeling within 4 weeks after ALND [26]. Since postoperative RT is typically initiated around 6 weeks after surgery—or later if adjuvant chemotherapy is given—these collateral networks are likely already established at the time of RT. This temporal and anatomical variability suggests that a pre-defined, static OAR such as the ALTJ may not fully represent the actual high-risk lymphatic drainage regions postoperatively. Thus, future research is needed to integrate postoperative lymphatic imaging, such as RT-planning-time lymphoscintigraphy, to map valid arm-draining LNs for each patient.

Such a clinical study was once performed by Cheville et al., who demonstrated the feasibility of integrating Single-Photon Emission Computed Tomography-Computed Tomography lymphoscintigraphy into breast cancer RT planning to identify and spare LNs critical for arm drainage [27]. The modified treatment plans resulted in a significant reduction in mean radiation exposure to these LNs, from 23.6 Gy in the standard plans to 7.7 Gy. All 28 participants who received RT with the modified plans did not develop lymphedema. However, a limitation of this study is that these patients had early-stage breast cancer and 93% underwent sentinel LN biopsy. All patients received whole breast irradiation without RNI. In this patient group, the risk of lymphedema is already considered very low, even without a modified plan, which may limit the generalizability of the findings to higher-risk populations who require RNI.

This study has several limitations. First, our analysis of the 10 patients already revealed a clear, distinct, and highly statistically significant difference in the dose delivered to the ALTJ between the two planning methods, robustly confirming dosimetric feasibility; however, a prospective clinical study enrolling a larger cohort of patients is warranted to assess whether this selective axilla-sparing RNI can effectively reduce the incidence of lymphedema without compromising oncologic safety. Second, regarding fractionation, while WBI without RNI has been delivered using a hypofractionated scheme (42.4 Gy in 16 fractions) at our hospital, RNI in the present study was conducted using a conventional fractionation schedule. As hypofractionation is becoming the standard also for RNI, there is a recognized necessity for developing a robust consensus on the corresponding ALTJ dose-volume constraints. Third, the lack of cross-checks to assess interobserver variability for ALTJ contouring is another limitation of this study. Lastly, we acknowledge that the left anterior descending coronary artery was not individually contoured, though we anticipate its dose impact is likely negligible as the whole heart was contoured and analyzed.

## 5. Conclusions

The present study demonstrates the dosimetric feasibility of Tomotherapy-based selective axillary sparing, specifically the protection of the ALTJ, in breast cancer patients after ALND. This is especially valuable when the dissected axilla is excluded from RNI, as it enables the prevention of incidental irradiation in this region without negatively impacting the sparing of other critical organs.

## Figures and Tables

**Table 1:** Dosimetric parameters of ALTJ and AXL1.

	ALTJ			AXL1		
Parameters	Plan A	Plan B	*p*	Plan A	Plan B	*p*
V20Gy (%)	100 ± 0	90.7 ± 9.3	0.018	97.2 ± 3.4	94.9 ± 4.7	0.011
V30Gy (%)	93.1 ± 7.1	28.5 ± 5.6	0.005	92.1 ± 6.6	75.6 ± 5.8	0.005
V35Gy (%)	82.7 ± 13.5	9.6 ± 4.7	0.005	87.5 ± 7.6	64.5 ± 6.2	0.005
V40Gy (%)	67.7 ± 18.0	2.6 ± 2.5	0.005	79.7 ± 7.3	50.2 ± 6.9	0.005
Dmean (Gy)	41.7 ± 3.4	27.2 ± 1.3	0.005	43.9 ± 2.0	37.7 ± 1.9	0.005
Dmin (Gy)	29.5 ± 5.9	18.0 ± 2.2	0.005	14.5 ± 6.5	13.0 ± 4.6	0.201
Dmax (Gy)	51.4 ± 2.1	44.0 ± 4.2	0.005	53.8 ± 0.9	54.1 ± 0.6	0.182

ALTJ = axillary lateral vessel thoracic junction, AXL1 = axillary lymph node level I, Plan A = conventional plan, Plan B = study plan.

**Table 2:** Dosimetric parameters of standard OARs.

OAR	Parameters	Plan A	Plan B	*p*
Ipsilateral Lung	V20Gy (%)	22.3 ± 5.9	22.2 ± 5.6	0.233
Heart	V5Gy (%)	60.1 ± 14.4	57.2 ± 13.9	0.012
	V30Gy (%)	2.6 ± 2.5	2.1 ± 2.3	0.043
	Dmean (Gy)	4.8 ± 1.7	4.8 ± 1.6	0.336

OAR = organ at risk, Plan A = conventional plan, Plan B = study plan.

## Data Availability

The data from this study are available from the corresponding author upon request, subject to ethical considerations.
